# Supplementary material for: Bayesian refinement of protein structures and ensembles against SAXS data using molecular dynamics
Source: PLoS Comput Biol. 2017 Oct 18;13(10):e1005800. doi: 10.1371/journal.pcbi.1005800 (PMC5662244; doi:10.1371/journal.pcbi.1005800)
Supplement: S1 Archive — (BZ2) [file pcbi.1005800.s011.tar.bz2 › gromacs_4.62_bayesian_saxs_refi/share/html/online/editconf.html]

editconf


| editconf Main Table of Contents |

**VERSION 4.6  
Sat 19 Jan 2013**

---

### Description

editconf converts generic structure format to .gro, .g96
or .pdb.

The box can be modified with options -box, -d and
-angles. Both -box and -d
will center the system in the box, unless -noc is used.

Option -bt determines the box type: triclinic is a
triclinic box, cubic is a rectangular box with all sides equal
dodecahedron represents a rhombic dodecahedron and
octahedron is a truncated octahedron.
The last two are special cases of a triclinic box.
The length of the three box vectors of the truncated octahedron is the
shortest distance between two opposite hexagons.
Relative to a cubic box with some periodic image distance, the volume of a
dodecahedron with this same periodic distance is 0.71 times that of the cube,
and that of a truncated octahedron is 0.77 times.

Option -box requires only
one value for a cubic, rhombic dodecahedral, or truncated octahedral box.

With -d and a triclinic box the size of the system in the x-, y-,
and z-directions is used. With -d and cubic,
dodecahedron or octahedron boxes, the dimensions are set
to the diameter of the system (largest distance between atoms) plus twice
the specified distance.

Option -angles is only meaningful with option -box and
a triclinic box and cannot be used with option -d.

When -n or -ndef is set, a group
can be selected for calculating the size and the geometric center,
otherwise the whole system is used.

-rotate rotates the coordinates and velocities.

-princ aligns the principal axes of the system along the
coordinate axes, with the longest axis aligned with the x-axis.
This may allow you to decrease the box volume,
but beware that molecules can rotate significantly in a nanosecond.

Scaling is applied before any of the other operations are
performed. Boxes and coordinates can be scaled to give a certain density (option
-density). Note that this may be inaccurate in case a .gro
file is given as input. A special feature of the scaling option is that when the
factor -1 is given in one dimension, one obtains a mirror image,
mirrored in one of the planes. When one uses -1 in three dimensions,
a point-mirror image is obtained.

Groups are selected after all operations have been applied.

Periodicity can be removed in a crude manner.
It is important that the box vectors at the bottom of your input file
are correct when the periodicity is to be removed.

When writing .pdb files, B-factors can be
added with the -bf option. B-factors are read
from a file with with following format: first line states number of
entries in the file, next lines state an index
followed by a B-factor. The B-factors will be attached per residue
unless an index is larger than the number of residues or unless the
-atom option is set. Obviously, any type of numeric data can
be added instead of B-factors. -legend will produce
a row of CA atoms with B-factors ranging from the minimum to the
maximum value found, effectively making a legend for viewing.

With the option -mead a special .pdb (.pqr)
file for the MEAD electrostatics
program (Poisson-Boltzmann solver) can be made. A further prerequisite
is that the input file is a run input file.
The B-factor field is then filled with the Van der Waals radius
of the atoms while the occupancy field will hold the charge.

The option -grasp is similar, but it puts the charges in the B-factor
and the radius in the occupancy.

Option -align allows alignment
of the principal axis of a specified group against the given vector,
with an optional center of rotation specified by -aligncenter.

Finally, with option -label, editconf can add a chain identifier
to a .pdb file, which can be useful for analysis with e.g. Rasmol.

To convert a truncated octrahedron file produced by a package which uses
a cubic box with the corners cut off (such as GROMOS), use:  
editconf -f in -rotate 0 45 35.264 -bt o -box veclen -o out  
where veclen is the size of the cubic box times sqrt(3)/2.

### Files

| option | filename | type | description |
| --- | --- | --- | --- |
| **-f** | conf.gro | Input | Structure file: gro g96 pdb tpr etc. |
| **-n** | index.ndx | Input, Opt. | Index file |
| **-o** | out.gro | Output, Opt. | Structure file: gro g96 pdb etc. |
| **-mead** | mead.pqr | Output, Opt. | Coordinate file for MEAD |
| **-bf** | bfact.dat | Input, Opt. | Generic data file |

### Other options

| option | type | default | description |
| --- | --- | --- | --- |
| **-[no]h** | bool | no | Print help info and quit || **-[no]version** | bool | no | Print version info and quit || **-nice** | int | 0 | Set the nicelevel || **-[no]w** | bool | no | View output .xvg, .xpm, .eps and .pdb files || **-[no]ndef** | bool | no | Choose output from default index groups || **-bt** | enum | triclinic | Box type for -box and -d: triclinic, cubic, dodecahedron or octahedron || **-box** | vector | 0 0 0 | Box vector lengths (a,b,c) || **-angles** | vector | 90 90 90 | Angles between the box vectors (bc,ac,ab) || **-d** | real | 0 | Distance between the solute and the box || **-[no]c** | bool | no | Center molecule in box (implied by -box and -d) || **-center** | vector | 0 0 0 | Coordinates of geometrical center || **-aligncenter** | vector | 0 0 0 | Center of rotation for alignment || **-align** | vector | 0 0 0 | Align to target vector || **-translate** | vector | 0 0 0 | Translation || **-rotate** | vector | 0 0 0 | Rotation around the X, Y and Z axes in degrees || **-[no]princ** | bool | no | Orient molecule(s) along their principal axes || **-scale** | vector | 1 1 1 | Scaling factor || **-density** | real | 1000 | Density (g/L) of the output box achieved by scaling || **-[no]pbc** | bool | no | Remove the periodicity (make molecule whole again) || **-resnr** | int | -1 | Renumber residues starting from resnr || **-[no]grasp** | bool | no | Store the charge of the atom in the B-factor field and the radius of the atom in the occupancy field || **-rvdw** | real | 0.12 | Default Van der Waals radius (in nm) if one can not be found in the database or if no parameters are present in the topology file || **-[no]sig56** | bool | no | Use rmin/2 (minimum in the Van der Waals potential) rather than σ/2 || **-[no]vdwread** | bool | no | Read the Van der Waals radii from the file vdwradii.dat rather than computing the radii based on the force field || **-[no]atom** | bool | no | Force B-factor attachment per atom || **-[no]legend** | bool | no | Make B-factor legend || **-label** | string | A | Add chain label for all residues || **-[no]conect** | bool | no | Add CONECT records to a .pdb file when written. Can only be done when a topology is present |

### Known problems

- For complex molecules, the periodicity removal routine may break down, in that case you can use trjconv.

---

http://www.gromacs.org  
gromacs@gromacs.org
